# *KIRREL3*-related disorders: a case report confirming the radiological features and expanding the clinical spectrum to a less severe phenotype

**DOI:** 10.1186/s13052-023-01488-7

**Published:** 2023-08-21

**Authors:** Andrea Querzani, Fabio Sirchia, Gianluca Rustioni, Alessandra Rossi, Alessandro Orsini, Gian Luigi Marseglia, Salvatore Savasta, Luisa Chiapparini, Thomas Foiadelli

**Affiliations:** 1 Pediatric Clinic, Fondazione IRCCS Policlinico San Matteo, Pavia, PV Italy; 2 Department of Molecular Medicine, University of Pavia, Pavia, 27100 Italy; 3 Medical Genetics Unit, IRCCS San Matteo Foundation, Pavia, Italy; 4 Pediatric Neurology, University Hospital of Pisa, Azienda Ospedaliero Universitaria Pisana, Pisa, 56126 Italy; 5 Pediatric Clinic and Rare Diseases, P.O. Pediatrico Microcitemico “A. Cao”, Università degli Studi di Cagliari, Cagliari, CA Italy; 6 Department of Neuroradiology, Fondazione IRCCS Policlinico San Matteo, Pavia, PV Italy

**Keywords:** KIRREL3, Neurodevelopmental disorders, Cerebellar hypoplasia, CASK, Case report

## Abstract

**Background:**

Neurodevelopmental disorders have a multifactorial etiology, since biological, genetic, psychosocial and environmental risk factors are involved. Recent studies have been linking neurodevelopmental disorders and intellectual disability with a variety of genes, some of which encoding neuronal cell-adhesion molecules. Among these, KIRREL3 is known to play a role in CNS development, and his variants have recently been related to intellectual disability, autism spectrum disorder, childhood apraxia of speech, cerebellar hypoplasia and mild dysmorphic features.

**Case presentation:**

In this study, we describe a young Caucasian boy with mild intellectual disability, cerebellar anomalies (cerebellar hypoplasia and mega cisterna magna) and minor dysmorphic features associated to a novel *KIRREL3* variant.

**Conclusions:**

Aim of the present case report is to expand the clinical spectrum of *KIRREL3*-related diseases towards a milder phenotype than what is already described in the literature. We speculate that the interaction between KIRREL3 and CASK might play a major role in promoting cognitive and cerebellar development, contributing to a variety of clinical manifestations.

**Supplementary Information:**

The online version contains supplementary material available at 10.1186/s13052-023-01488-7.

## Background

Neurodevelopmental disorders cover a wide spectrum of conditions related to neurological system and brain; they include autism spectrum disorder (ASD), attention-deficit/hyperactivity disorder (ADHD), intellectual disability (ID), learning disability and many others [1]. These conditions have a high inter- and intra-individual variability, as children affected by the same condition may have different clinical presentations, and symptoms can vary greatly over time even in the same patient. The majority of neurodevelopmental disorders can be linked to a multifactorial etiology, since biological, genetic, psychosocial and environmental risk factors can play a role. However, only in less than 50% of cases a precise cause is found, especially when the impairment is mild [2]. In the last decades, a modern diagnostic approach to neurodevelopmental disorders and the use of genetic testing such as Next Generation Sequencing (NGS) are increasingly leading to the identification of novel genes in a growing number of individuals, contributing to the definition of previously uncharacterized clinical patterns [2]. Nevertheless, most developmental disabilities still don’t have any acknowledged etiology nowadays [3].

Recent studies have been linking neurodevelopmental disorders and ID with a variety of genes encoding neuronal cell-adhesion molecules, which are involved in neuronal network development, cell migration, axonal guidance, synapse formation and synaptic plasticity [4]. The remarkable advances in the field of mental retardation (MR) allow to shed light on how defects in synaptogenesis and synaptic plasticity are crucial processes in determining intellectual development [2]. As observed in monogenic causes of MR and especially in the Fragile X Syndrome, morphological and/or functional abnormalities of synapses in the cerebral cortex, cerebellum and hippocampus have been shown to contribute to determine cognitive deficit [2, 5].

The kin of irregular chiasm-like proteins (KIRREL, also known as Kirre like nephrin family adhesion molecule) are cell-adhesion molecules of the immunoglobulin (Ig) superfamily characterized by three type I membrane proteins with five extracellular Ig-like domains: KIRREL1, KIRREL2 and KIRREL3^3,4,6^.

*KIRREL3* (OMIM #607,761) is located on the long arm of chromosome 11 (11q24.2), and encodes for a synaptic cell adhesion molecule involved in neuronal migration, axonal fasciculation and synaptogenesis [6]. The highest expression of *KIRREL3* is found in neurons of the central nervous system (CNS), particularly in the olfactory, limbic, auditory and cerebellar circuits [4]. Even if its expression pattern is still to be completely defined, *KIRREL3* is known to play a role in CNS development, contributing to cell recognition and being involved in the nucleogenesis of the pontine nucleus through the control of neuronal migration [7]. *KIRREL3* variants have recently been related to ID, ASD, childhood apraxia of speech, cerebellar hypoplasia and mild dysmorphic features [4]. In addition, *KIRREL3* was recognized as one of the strongest gene candidates in causing Jacobsen syndrome, a contiguous gene deletion syndrome where ID and ASD are distinctive features along with skeletal malformations and dysmorphisms [8]. Finally, some authors reported an association between behavioral anomalies and KIRREL3 deficiency also in mice models [4].

We hereby wish to expand the clinical spectrum of KIRREL3-associated disease by describing the case of a young boy with a milder phenotype, supporting the role of KIRREL3 in cerebellar formation and function, further speculating on the possible underlying molecular mechanisms.

## Case Presentation

**Fig. 1:**
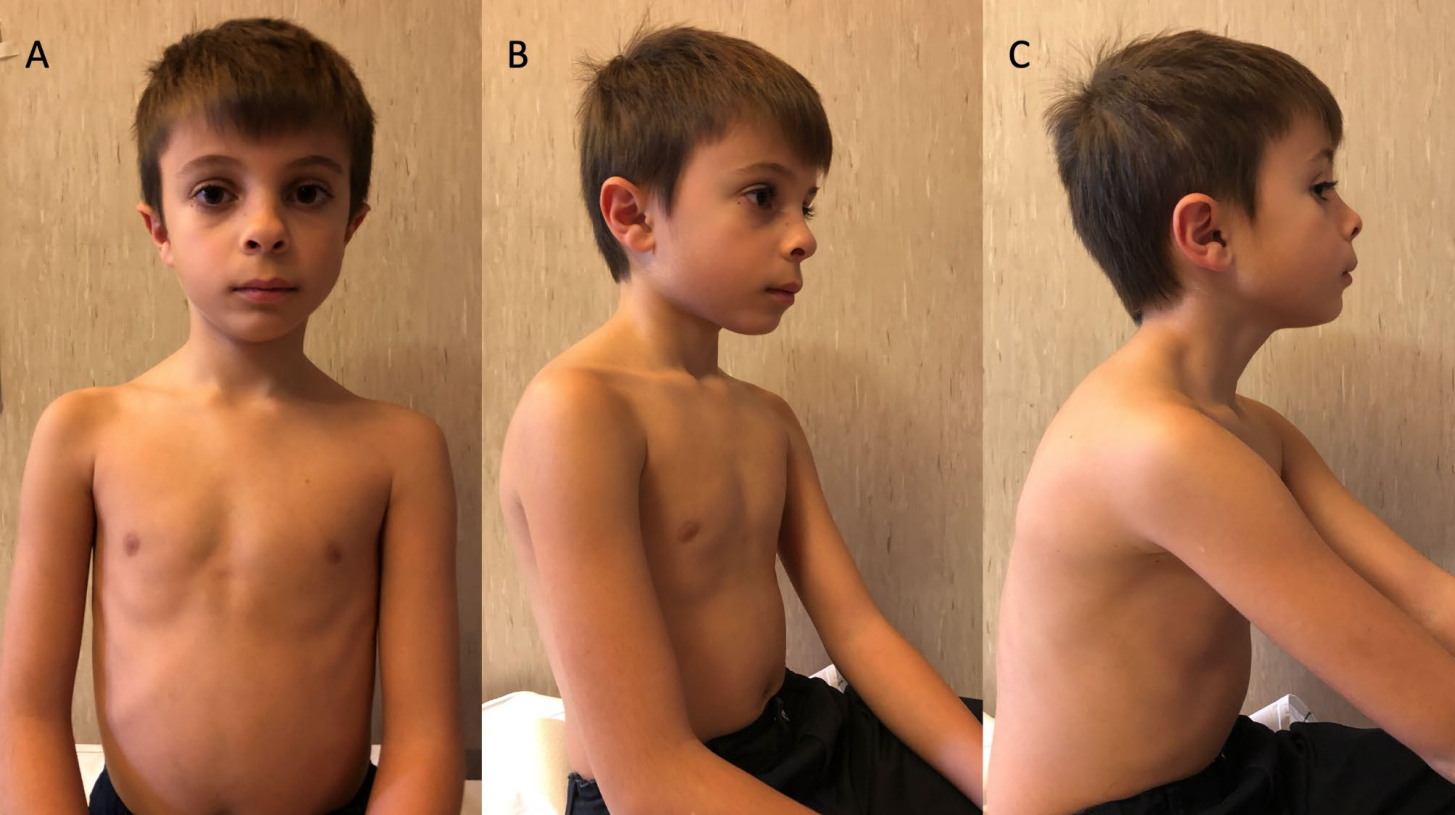
Clinical phenotype at 11, with minor dysmorphic features: long eyelashes, arched eyebrows, slightly anteverted nostrils, long nasal philtrum, mild asymmetric pectus excavatum

A 4-years-old Caucasian male came to our attention for persistent asymptomatic elevation of serum creatine-phosphokinase (CPK) levels. He was born at term by cesarean delivery after a pregnancy complicated by maternal hypertension and hypothyroidism. Family history was unremarkable. At birth, the patient was hospitalized in the NICU for neonatal sepsis complicated by hypoglycemia. Early cognitive development was mildly affected, with a delayed acquirement of the main language milestones (lallation at 12 months, first words at 18 months, simple sentences at 30 months), with normal motor development. At the time of evaluation, CPKs were found to be elevated in various samples over several months (up to twice the normal value, range 219–315 mg/dl), with concurrent elevation in serum lactate dehydrogenase and aldolase. No muscle symptoms such as fatigue, myalgia, exercise intolerance or gait alterations were reported. Physical examination was normal, except for mild dysmorphic features: long eyelashes, slightly anteverted nostrils, long nasal philtrum, mild asymmetric pectus excavatum (Fig. 1). His fine and gross motor skills were preserved, and Gowers sign was negative. Auxologic parameters were on the low centiles (3-10th centile) according to sex and age, fitting the mid parental target height. Limb electromyography, EKG and cardiac ultrasound were normal. A Next-Generation Sequencing (NGS) gene panel for hyper-CPKemia revealed a variant of uncertain significance on *PYGM*, inherited from the healthy father (who had normal CPK values) and classified as likely benign. The assessment of global cognitive functioning, performed with the Wechsler Intelligence Scale for Children, 4th edition (WISC-IV) [9] and repeated at 6 and 9 years of age, showed worsening verbal comprehension skills (Verbal Comprehension Index declining from 80 to 64 – defective), with markedly improving working memory (Working Memory Index from 82 to 103). Fluid reasoning (Fluid Reasoning Index from 82 to 87) and processing speed (Processing Speed Index from 82 to 85) scores remained in the low normal range over time, deriving an overall borderline Intelligence Quotient (IQ: 75–77). He attended primary school with a dedicated teacher for special educational needs, received pedagogical support for homework and attended weekly psychomotricity sessions.

**Fig. 2:**
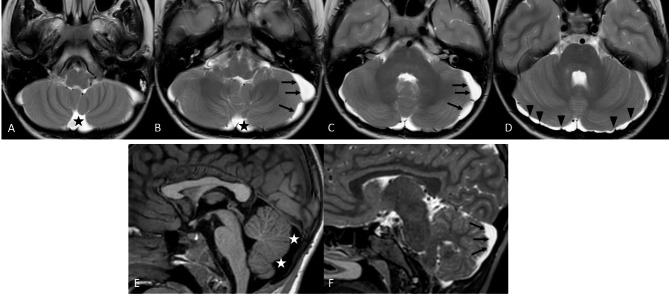
Brain MRI. Axial and parasagittal Sagittal T2wi (**A-D** and **F**) and Sagittal T1wi (**E**) demonstrate mega cisterna magna (asterisk) associated with mild cerebellar hemispheres hypoplasia (arrows) and typical splitting of the cerebellar falx (arrowheads)

At 9 years old, brain MRI showed a moderate enlargement of the peri-cerebellar CFS spaces due to mild cerebellar hypoplasia, mega cisterna magna and a splitting of the cerebellar falx (Fig. 2). A trio whole exome sequencing (WES) was performed, revealing a novel *de novo* heterozygous missense variant in *KIRREL3* (NM_032531.4) [c.2003G > C; p.(Arg668Pro)]. The pathogenicity of this variant was assessed by using bioinformatic tools [8, 10], by its frequency in gnomAD v2.1.1 (Absent from controls in Exome Sequencing Project, 1000 Genomes Project, or Exome Aggregation Consortium) [9] and by its de novo origin. The KIRREL3 variant was thus classified as hot VUS (a variant of uncertain significance which may become pathogenic with additional proof of pathogenicity) according to ACMG criteria (PP4, PS2, PM2) [10]. Clinical follow up was continued until the age of 11 years, annual CPK dosages showed progressive normalization, without new neuro-muscular signs or symptoms.

## Discussion and conclusions

Including the present report, only 21 individuals with neurodevelopmental disorder and *KIRREL3* variants have been described so far (Table 1) [3, 10–17]. Most of genetic alterations are missense mutations, while two cases present chromosomal translocations involving chromosome 11. All of them had different degrees of developmental delay and/or ID, ASD, attention deficit/hyperactivity disorder and mild non-gestaltic dysmorphic features [4, 10].

**Table 1:** KIRREL3 alterations described in literature to date

PATIENT	STUDY	KIRREL3 ALTERATION	INHERITANCE	CLINICAL PRESENTATION
**1**	Bhalla et al. [3] (2008)	c.2191G > T; p.Val73Phe	*De novo*	Mild ID
**2**	Bhalla et al. [3] (2008)	c.1007G > A; p.Arg336Gln	*De novo*	ID
**3**	Bhalla et al. [3] (2008)	c.1007G > A; p.Arg336Gln	*De novo*	ID
**4**	Bhalla et al. [3] (2008)	c.1007G > A; p.Arg336Gln	*De novo*	ID
**5**	Bhalla et al. [3] (2008)	c.118 C > T; p.Arg40Trp	*De novo*	Severe ID
**6**	Bhalla et al. [3] (2008)	t(11;16)(q24.2;q24) involving KIRREL3	*De novo*	Severe ID, dysmorphisms
**7**	Talkowski et al. [11] (2012)	t(11;X)(q24.2;p11.4) involving KIRREL3	*De novo*	Developmental disorder
**8**	Michaelson et al. [12] (2018)	chr11:126293122 A > G (non coding)	*De novo*	ASD
**9**	Michaelson et al. [12] (2018)	chr11:126293122 A > G (non coding)	*De novo*	ASD
**10**	Kalsner et al. [14] (2018)	c.1177G > A p.Ala393Thr	Maternal	DD, ASD, dysmorphisms, Chiari I malformation
**11**	Kalsner et al. [14] (2018)	c.1238 C > T p.Thr413Ile	Paternal	DD, ASD, dysmorphisms, Chiari I malformation
**12**	Kalsner et al. [14] (2018)	c.1177G > A p.Ala393Thr	Maternal	DD, ASD, dysmorphisms, central apnea
**13**	Kalsner et al. [14] (2018)	c.1231G > C p.Glu411Gln	Maternal	DD, ASD, dysmorphisms, allergies
**14**	Kalsner et al. [14] (2018)	c.1566G > T p.Lys522Asn	Paternal	DD, ASD
**15**	Kalsner et al. [14] (2018)	c.655G > T p.Val219Leu	Paternal	DD, ASD
**16**	Leblond et al. [15] (2019)	c.1685G > T p.Arg562Leu	Maternal and Paternal (homozygosity)	ASD
**17**	Guo et al. [16] (2019)	c. 1985G > A p.Arg662His	*De novo*	ASD
**18**	Taylor et al. [13] (2020)	c.2019G > A p.Met673Ile	*De novo*	ID, ADHD, obesity, dysmorphisms, altered fine motility, brain MRI alterations
**19**	Hildebrand et al. [17] (2020)	c.2186G > T p.Ser729Ile	Unknown	CAS, phonological delay, reading and spelling deficits, speech pathology, dysmorphisms
**20**	Ciaccio et al. [10] (2021)	c.764 C > T p.Ser255Leu	*De novo*	ID, ASD, muscular hypotonia, ataxic gate, EEG abnormalities, dysmorphisms, brain MRI alterations
**21**	Present study	c.2003G > C; p.Arg668Pro	*De novo*	Mild ID, dysmorphisms, brain MRI alterations

Abbreviations: ID = intellectual disability; ASD = autism spectrum disorder; DD = developmental delay; ADHD = attention deficit/hyperactivity disorder; MRI = magnetic resonance imaging; CAS = childhood apraxia of speech; EEG = electroencephalogram.

Our patient’s mutation, together with his clinical history, can represent an additional demonstration of the pathogenic role of KIRREL3. He underwent brain MRI at the age of 9, which showed enlarged pericerebellar liquoral spaces bilaterally. MRI has been performed in few of the patients with *KIRREL3* pathogenic variants, reported in Table 1. In particular, the MRI performed on patients 10 and patient 11 showed Chiari I malformation. Patient 18 had mild temporal cortical atrophy with wide asymmetric posterior ventricular horns, while patient 20 presented mild cerebellar hypoplasia and mega cisterna magna that was very similar to our patient’s neuroradiological phenotype. Comparable cerebellar abnormalities were also reported by Puvabanditsin et al. [8] in a patient affected by Jacobsen syndrome presenting hypoplasia of cerebellar vermis, enlarged cisterna magna, bilateral ventriculomegaly and residuals of germinal matrix hemorrhage.

Interestingly, KIRREL3 interacts and colocalizes with CASK (calmodulin associated serine/threonine kinase, located on Xp11.4, OMIM #300,172) [3] in human neuronal cells. Immunohistochemical studies carried on by Gerke et al. revealed specific interactions between KIRREL3 and the PDZ domain of CASK [6]. As a synaptic scaffolding protein, it functions as part of large signaling complexes in both pre- and postsynaptic sites [18], and his deletion in mice has been related to impaired synaptic function [3]. By interacting with the brain-specific T-box transcription factor TBR1 and enhancing his transcriptional activity, CASK regulates the expression of the protein Reelin, a key player in neuronal migration and lamination [18]. Furthermore, *CASK* variants have been widely described in correlation with microcephaly and cerebellar hypoplasia [19].

In 2021, Ciaccio and colleagues [10] were the first to deepen the radiological features of cerebellar hypoplasia and mega cisterna magna, speculating on the possible underlying involvement of CASK-associated molecular pathway. They reported a boy with mild facial dysmorphism, generalized muscle hypotonia, delayed motor and language milestones, attention deficit and emotional dysregulation. Electroencephalography showed sparse multifocal epileptic anomalies, whereas brain MRI revealed a mega cisterna magna with mild cerebellar hypoplasia. While their patient had severe ID and gait alterations [10], our case presented with only borderline intellectual ability and mild language delay. We believe that the presented case strengthens the clinical and neuroradiological findings already proposed by Ciaccio et al., and the possible KIRREL3-CASK interaction in promoting cognitive and cerebellar development.

While the clinical features of *KIRREL3*-related disease are currently mainly defined by various degrees of neurodevelopmental disorders, milder phenotypes with normal-borderline cognitive abilities and minor cerebellar anomalies might emerge in the future, representing new and more difficult challenges in the diagnostic process.

### Electronic supplementary material

Below is the link to the electronic supplementary material.

Supplementary Material 1

## Data Availability

All data that support the findings of this study are available from the corresponding author, upon reasonable request.

## List of abbreviations

ADHD: Attention deficit/hyperactivity disorder
ASD: Autism spectrum disorder
CAS: Childhood apraxia of speech
CNS: Central nervous system
CPK: Creatine-phosphokinase
DD: Developmental delay
EEG: Electroencephalogram
ID: Intellectual disability
MR: Mental retardation
MRI: Magnetic resonance imaging
NGS: Next generation sequencing
NICU: Neonatal intensive care unit
VUS: Variant of uncertain significance
WES: Whole exome sequencing
